# Pyrotechnic Delay Compositions Based on BaO_2_: Not as “Green” as Expected

**DOI:** 10.3390/molecules28166143

**Published:** 2023-08-19

**Authors:** Kinga Lysien, Klaudia Szatan, Konrad Szydlo, Mateusz Polis, Marcin Procek, Agnieszka Stolarczyk, Tomasz Jarosz

**Affiliations:** 1 Department of Physical Chemistry and Technology of Polymers, Silesian University of Technology, 44-100 Gliwice, Polandagnieszka.stolarczyk@polsl.pl (A.S.); 2 Faculty of Chemistry, Silesian University of Technology, 44-100 Gliwice, Poland; 3 Explosive Techniques Research Group, Łukasiewicz Research Network—Institute of Industrial Organic Chemistry, 42-693 Krupski Młyn, Poland; konrad.szydlo@ipo.lukasiewicz.gov.pl (K.S.); mateusz.polis@ipo.lukasiewicz.gov.pl (M.P.); 4 Department of Optoelectronics, Silesian University of Technology, 2 Krzywoustego Str., 44-100 Gliwice, Poland; marcin.procek@polsl.pl

**Keywords:** pyrotechnic delay composition, combustion velocity, mechanism, barium peroxide, green pyrotechnics, combustion product

## Abstract

The aims of this study were to investigate the potential of using barium peroxide as an environmentally friendly oxidising agent, to evaluate the composition of the combustion products of the developed pyrotechnic delay compositions (PDCs) and to provide information about the impact of the utilised metallic fuel (Mg, Al, Fe or Cu) on the properties of those PDCs. The PDCs exhibited acceptable friction and impact sensitivity values. This allowed conducting further experiments, e.g., determining the linear combustion velocity of the PDCs as a function of oxygen balance (OB). Based on the composition of the post-combustion residues, determined by Raman spectroscopy and SEM-EDS, an initial mechanism for the combustion of the developed PDCs was proposed.

## 1. Introduction

Pyrotechnic delay compositions (PDCs), i.e., pyrotechnic compositions characterised by a highly stable combustion velocity and either low or lack of emission of gaseous combustion products, are extensively used in pyrotechnic devices, such as detonators, to introduce a time interval (“delay”) between the device receiving an initiating signal and being activated, thus activating, e.g., a primary explosive charge. The extensive use of PDCs in delay detonators [1] is the foundation of modern blasting operations. Careful selection of delays between the detonations of individual charges constituting a blasting grid is used to minimise the undesirable seismic effects of blasting operations on their environment, as well as achieving the desired fragmentation profile of the mined rock [2].

A broad variety of PDCs has been reported in the literature, with a significant increase in works dedicated to these systems in recent years [3,4,5]. Systems utilising numerous oxidising agents and fuels have been reported [6,7]. The traditional PDCs, i.e., Sb/KMnO_4_ and Si/Pb_3_O_4_, however, continue to remain in widespread use, despite the concerns over their adverse effect on human health and on the environment. This state of the art stems from the fact that the two traditional PDC formulations offer adequate performance, while being relatively inexpensive. The proposed alternative PDCs, utilising fuels such as titanium or zirconium, may exhibit better performance and even a negligible impact on human health and the environment, but these desirable features come at a disproportionately greater cost, making their widespread adoption unlikely.

In light of the above, the design of a “green” PDC needs to take into account energetic properties, impact on human health and on the environment, as well as economical factors. Of the viable choices for fuels, aluminium and iron are of particular interest, due to their low unit cost and low impact and their combustion products on human health and on the environment. Of the two fuels, aluminium exhibits a high heat of combustion (Table 1), whereas iron exhibits a vastly higher melting point, which allows the losses of energy associated with phase transitions to be minimised, even if contributing to a significantly lower heat of combustion. The other prospective fuels are relatively expensive (e.g., Ti), highly toxic (e.g., B, Be, Sb) or offer limited heats of combustion while having high oxygen consumption (e.g., Sb).

The choice of oxidising agent is more complex, as the heats of dissociation of these compounds are an order of magnitude lower than the heats of combustion of the fuels and do not differ significantly among themselves (Table 1). If the amount of oxygen that can be theoretically supplied by the oxidising agents is considered, molybdenum(VI) oxide would be of interest, but the compound has the drawback of significant toxicity [8].

**Table 1:** Thermodynamic data for selected fuels and oxidising agents.

Fuels				
Fuel	Melting Point [°C]	Heat of Combustion [kJ/g] ^a^	ΔO_2_ [mol/g] ^b^	Ref.
Mg	650	24.69	−2.057 × 10^−2^ (MgO)	[9]
Al	660	31.09	−5.559 × 10^−2^ (Al_2_O_3_)	[9,10]
Ti	1730/1666	15.86	−2.089 × 10^−2^ (TiO_2_)	[9,10]
Sb	630	2.89	−1.232 × 10^−2^ (Sb_2_O_3_)	[9]
B	2077	58.99	−1.388 × 10^−1^ (B_2_O_3_)	[9,10]
Be	1287	68.2	−5.548 × 10^−2^ (BeO)	[9,10]
Fe	1536	4.73	−2.686 × 10^−2^ (Fe_2_O_3_)	[9,10]
Cu	1082	2.46	−7.868·10^−3^ (CuO)	[11]
**Oxidising Agents**				
**Agent**	**Melting Point [°C]**	**Heat of Dissociation [kJ/g] ^c^**	**ΔO_2_ [mol/g] ^b^**	**Ref.**
CuO	decomposition	1.96	+6.286 × 10^−3^ (Cu)	[10]
Fe_2_O_3_	decomposition	5.17	+9.393 × 10^−3^ (Fe)	[10]
MoO_3_	802	5.18	+1.042 × 10^−2^ (Mo)	[10]
WO_3_	1473	3.64	+6.470 × 10^−3^ (W)	[10]
SnO_2_	1630	3.85	+6.635 × 10^−3^ (Sn)	[12,13]
BaO_2_	decomposition	3.75	+5.906 × 10^−3^ (Ba)	[14]

^a^ Recalculated from kcal/g, assuming 1 cal = 4.184 J; ^b^ Consumption of oxygen (negative sign) upon burning of a unit mass of the fuel or oxygen produced upon decomposition (positive sign) of a unit mass of the oxidising agent. Assumed reaction products are given in parentheses; ^c^ The heats of dissociation values listed are the negative of the heats of formation of the oxides. The values have been recalculated from kJ/mol.

In light of the above, barium peroxide may be an interesting choice of oxidising agent, as it can be produced directly via heating barium oxide at approximately 500–550 °C in the presence of air [15]. One significant drawback of this compound is the toxicity of the soluble compounds of barium [16]. Consequently, the use of this oxidising agent relies on the combustion products of the PDC containing solely insoluble barium compounds, such as the reported mixed oxides of barium and iron [17]. These reported compounds, however, have been produced by a mixture of solution precipitation and calcination rather than combustion, even though their formation was suggested in works dedicated to the combustion of the Fe/BaO_2_ PDC [18]. Mixed oxides of barium and aluminium have also been reported, but again, little information is available to predict whether they can be products of combustion for the Al/BaO_2_ PDC [19].

Consequently, the development of any PDCs containing barium peroxide requires an investigation into the nature of the combustion products and presence of soluble barium compounds, so as to ensure the “green” nature of these systems. In this work, we have presented the results of our investigations into the parameters of the Fe/BaO_2_ and Al/BaO_2_ PDCs, along with an initial study of the combustion products of those two systems, as well as two other prospective PDCs, i.e., Mg/BaO_2_ and Cu/BaO_2_.

## 2. Results and Discussion

### 2.1. Friction and Impact Sensitivity

The friction sensitivity of the two investigated PDCs (Table 2) is moderately high, as is common for pyrotechnic compositions containing metallic fuels [20]. Interestingly, the lowest friction sensitivity value (i.e., highest susceptibility to friction) is observed not for stoichiometric mixtures (OB = 0%), but for PDCs deficient in the oxidising agent. The Al/BaO_2_ in general shows lower friction sensitivity parameters than the Fe/BaO_2_, as expected due to the use of a more reactive metallic fuel.

The two PDCs are mostly insensitive to impact, with only Fe/BaO_2_ showing such sensitivity at an OB of −20%. The friction and impact sensitivity of the proposed PDCs is, therefore, within an acceptable margin, allowing further investigations on these systems to be conducted.

### 2.2. Linear Combustion Velocity

The Fe/BaO_2_ PDC shows low combustion velocities, achieving the highest velocity of 0.23 cm/s for a stoichiometric mixture (Table 3), as would be expected for a straightforward combustion mechanism. Even minor deviations (±5% OB) from this composition (translating into a Fe content change lesser than 10 wt.%) result in the observed combustion velocity being reduced to approximately half of this value. Further deviation from the stoichiometric composition results in even more drastic loss of combustion velocity. At an OB of −20%, the PDC sustains combustion, but the heat loss is so significant that the composition fails to achieve the temperature required to melt the copper probe wires.

Replacing iron with aluminium as the fuel results in a significant increase in the observed linear combustion velocity of the PDC by approximately one order of magnitude. Interestingly, the stoichiometric composition (OB = 0%) was characterised by the lowest observed linear combustion velocity. The highest combustion velocity is seen for a composition with an OB of +5%, which is out of the norm, as the composition would be expected to lose significant amounts of heat for the decomposition of the redundant oxidising agent molecules. The similar combustion velocities observed for compositions with OB values of −5% and −10% may be indicative of the presence of a local combustion velocity maximum. The co-occurrence of these two features may be indicative of the presence of two competing reactions underlying the combustion of Al/BaO_2_ PDCs.

The combustion of Mg/BaO_2_ took place extremely rapidly, with the observed linear combustion velocity being above the upper range of reliability for the utilised experimental set-up. In order to obtain reliable results, a different measurement method needs to be utilised, e.g., observation of the propagation of the combustion front by a high-speed camera. In the case of Cu/BaO_2_, the combustion was similar to that of Fe/BaO_2_ at an OB of −20% in that the wire probes used for measuring the combustion velocity were left intact, without breaking the circuit.

We have repeated the LCV measurements after compacting the PDC samples to approximately 30% TMD, in order to provide an initial insight into the effect of increased density on their combustion parameters. Samples with an oxygen balance of 0% were selected for this investigation and in this case, LCVs were reliably determined for all four investigated PDCs (Table 4). Interestingly, for the Fe/BaO_2_ PDC, compaction resulted in an increase in LCV, whereas in the case of the Al/BaO_2_ PDC, compaction translated to a decrease in LCV, in comparison with the uncompacted PDC samples. This can be related to the different gaseousness of the combustion processes of the two compositions. In case of Fe/BaO_2_, the reaction results in very low gaseousness. Once the material is compacted, the contact area increases, resulting in higher heat transfer coefficient. Due to the higher heat transfer coefficient, the combustion velocity also increase—the reaction in solid phase is driven mostly by heat exchange between solid components, via conduction. On the other hand, the combustion process of Al/BaO_2_ and Mg/BaO_2_ is characterised by very high gaseousness, and the main driving force is related to mass diffusion driven by gases and liquid phase movement. As the material is pressed and compacted, the number of free spaces decreases, thereby reducing the possibility of penetrating gases through the pores, which further accelerates the combustion process. Additionally, the initial temperature for such compositions usually exceeds the value of 700 °C. According to Table 1, the melting point for Mg and Al is lower that 700 °C, which can also be can be associated with the lower linear velocity after compaction. In the case of iron, the melting point significantly exceeds 700 °C.

### 2.3. Composition of PDC Combustion Products

#### 2.3.1. Scanning Electron Microscopy and X-ray Energy Dispersive Spectroscopy

**Figure A1:**
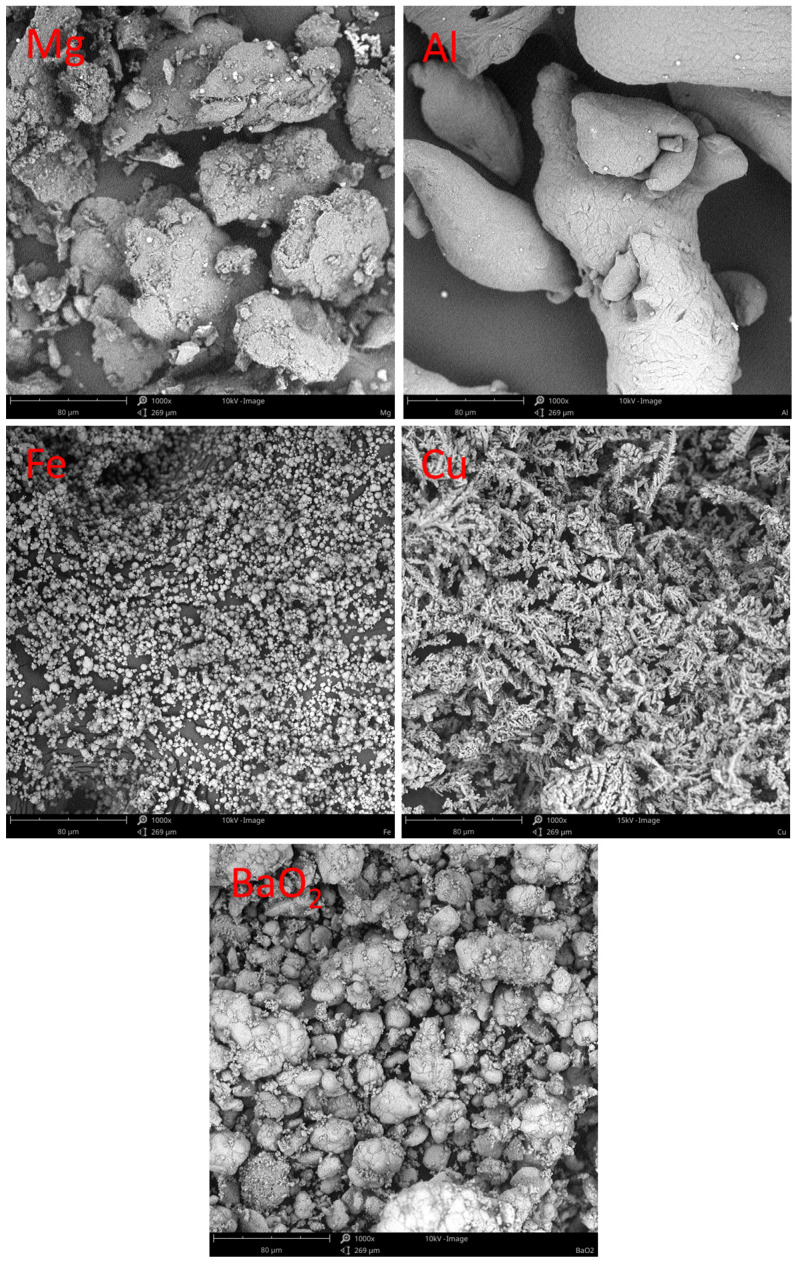
SEM images of the components used to produce the investigated PDCs.

The SEM images (Figure A1) acquired for the raw materials used for the preparation of the investigated PDCs reveal that each component has a distinct microstructure, with Cu having particularly well-developed particle shapes.

**Figure 1:**
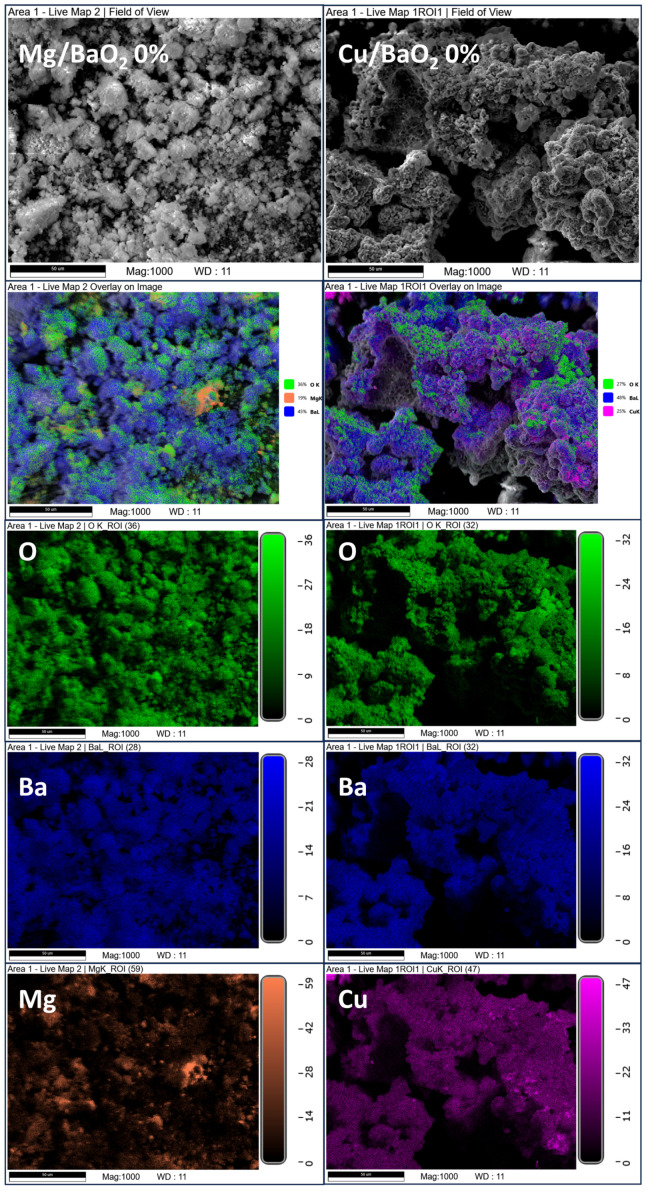
SEM images and EDS-based elemental mapping overlays for post-combustion residues of the Mg/BaO_2_ and Cu/BaO_2_ PDCs.

The SEM-EDS investigation of the Mg/BaO_2_ and Cu/BaO_2_ PDC post-combustion residues (Figure 1) reveals that the residues are covered to a very large extent in barium atoms, uniformly distributed over their surface. This is in line with the fact that achieving an OB of 0% for these compositions requires a BaO_2_ content in excess of 70 wt.%. However, the incidence of atoms originating from the utilised metallic fuels is remarkably low—in the case of Cu/BaO_2_, approximately 27 wt.% of copper is present in the PDC, but the EDS mapping reveals only several minor concentrations of this element in the sample. This suggests that upon combustion, the barium-bearing phase was pushed to the surface of the samples. This would be in line with the thermal decomposition of BaO_2_ and the evolving oxygen “foaming” the resulting barium oxide and residual barium peroxide.

**Figure 2:**
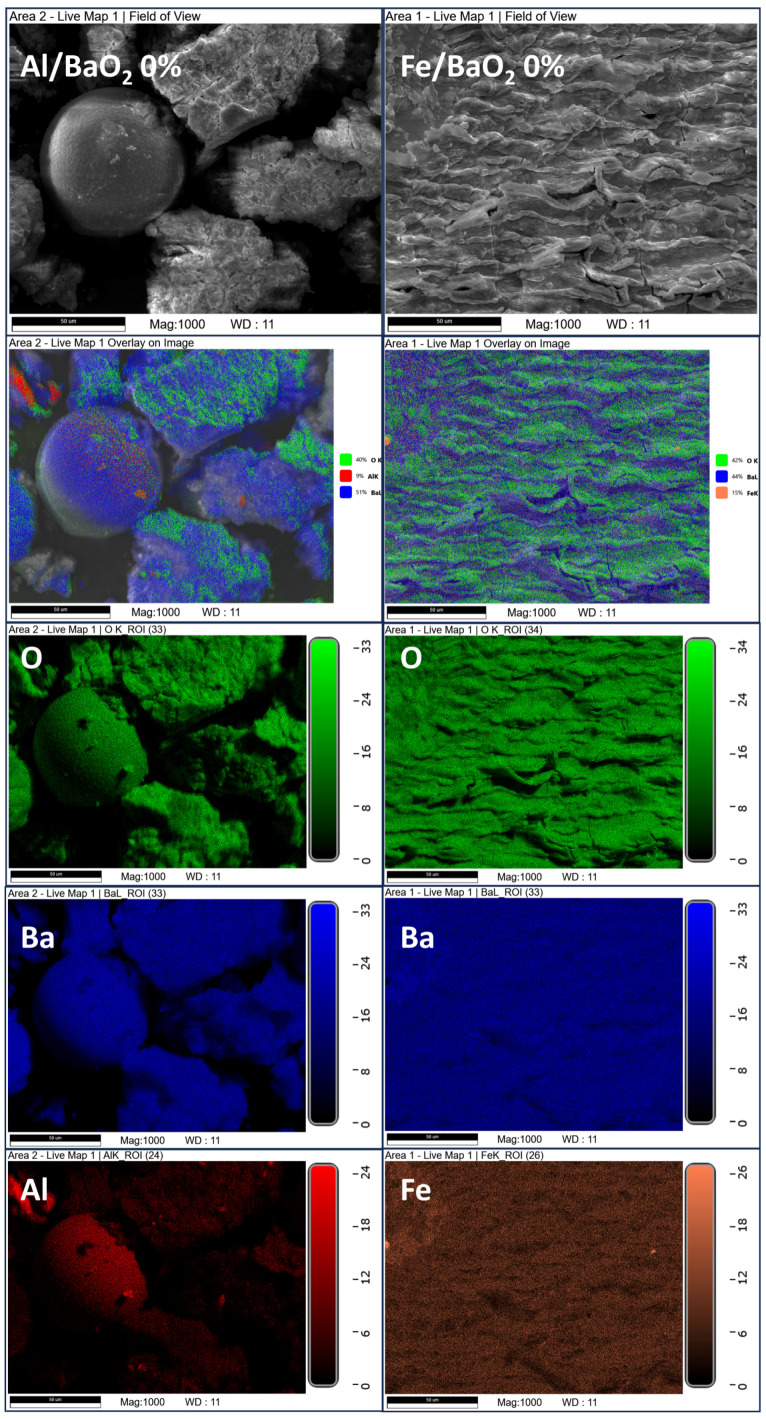
SEM images and EDS-based elemental mapping overlays for post-combustion residue of the Al/BaO_2_ and Fe/BaO_2_ PDCs.

In the case of the Fe/BaO_2_ PDCs (Figure 2), the PDCs, whose compositions correspond to OB values of +5, 0 and −5%, have their surface almost uniformly coated in barium, with no appreciable amounts of iron atoms being detected. This is the case even though the pure SEM image of the OB = −5% PDCs shows characteristic spherical agglomerates typical of the morphology of the utilised iron powder. As such, even though iron is present, it is covered by a layer of barium (likely in the form of barium oxide or barium peroxide). Only at lower OB values (−10 and −20%) can iron atoms be observed on the surface of the post-combustion residues. Even so, the distribution of iron atoms in the sample appears to be less widespread than the distribution of barium. In some cases, the two elements overlap to a significant extent, possibly indicative of the formation of mixed Ba-Fe oxides.

**Figure A4:**
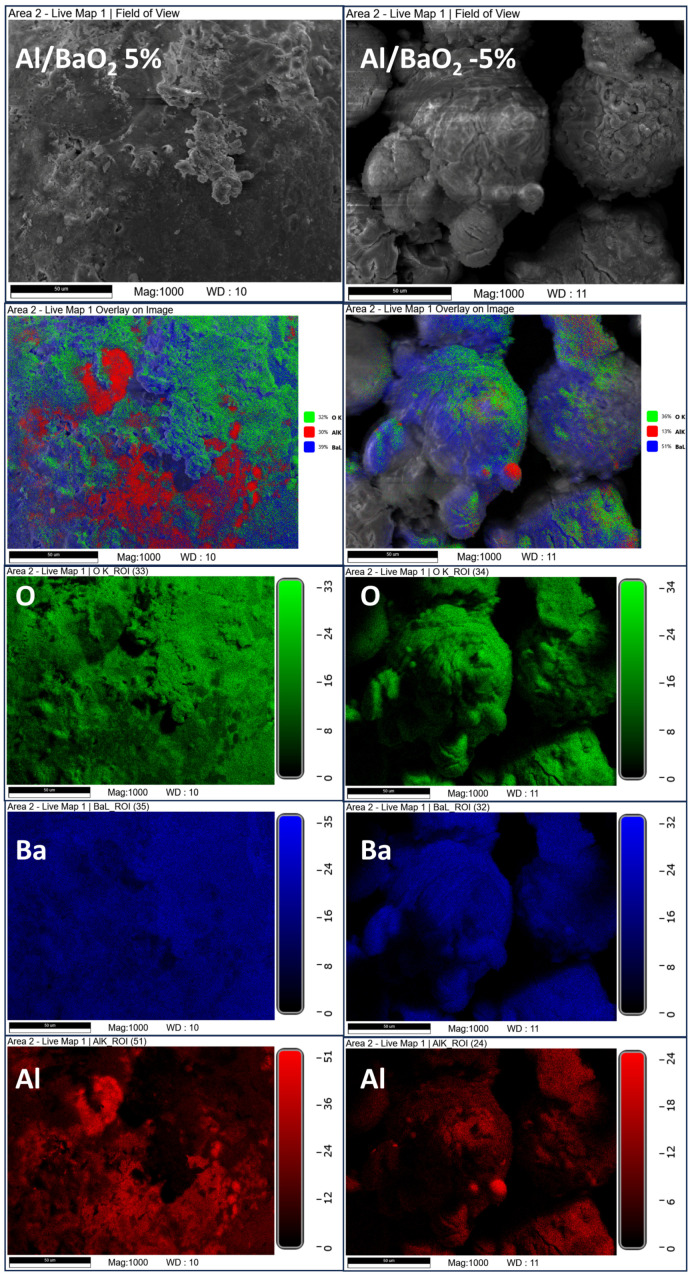
SEM images and EDS-based elemental mapping overlays for post-combustion residues of the Al/BaO_2_ PDC produced to have an oxygen balance of +5% and −5%.

**Figure A5:**
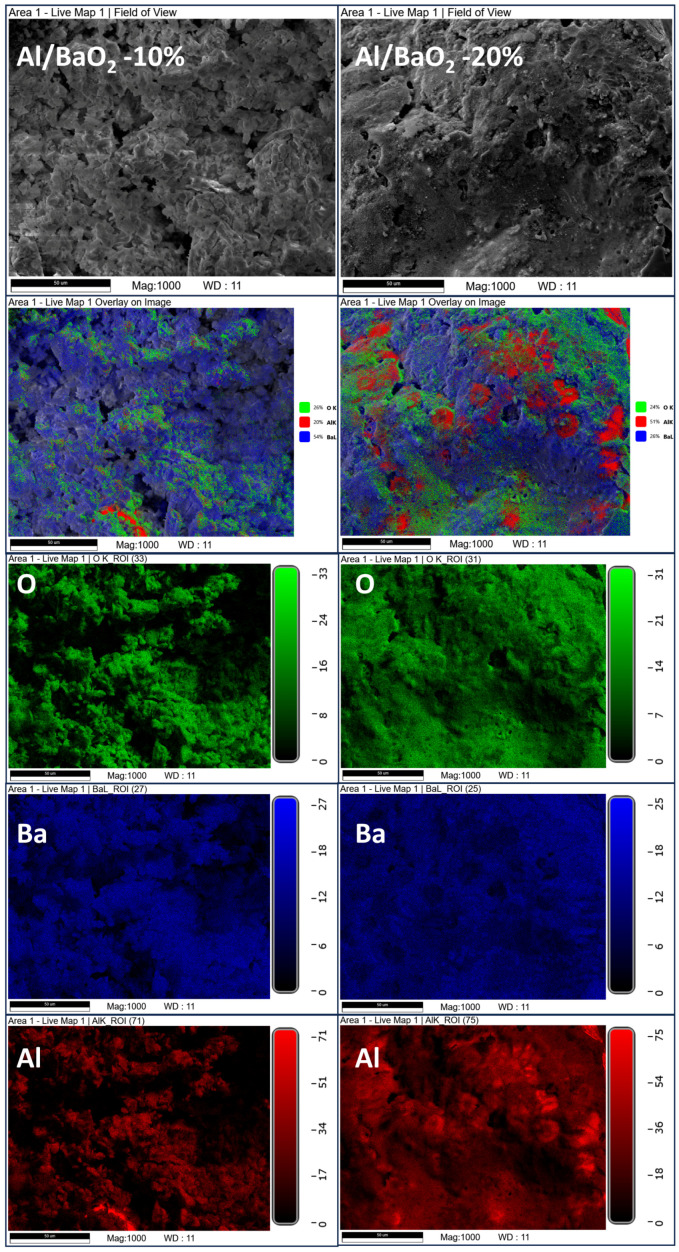
SEM images and EDS-based elemental mapping overlays for post-combustion residues of the Al/BaO_2_ PDC produced to have an oxygen balance of −10% and −20%.

The post-combustion residues produced using the Al/BaO_2_ PDCs (Figure A4 and Figure A5) show a practically identical distribution of barium and aluminium at OB values of −20–0%. In the case of the +5% OB post-combustion residue, the surface of the sample is coated with barium, much as in the case of the other barium-rich PDC samples. The similarity in the distribution of Ba and Al may be an indication of the formation of mixed Ba-Al oxides.

#### 2.3.2. Raman Spectroscopy

**Figure 3:**
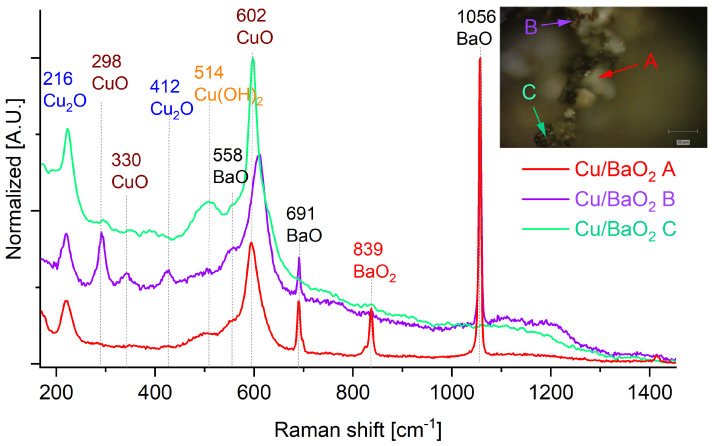
Raman spectra recorded for the combustion products of Cu/BaO_2_ PDC. Inset: Optical magnification of the sample with indicated points, for which Raman spectra were recorded.

Raman spectra of the post-combustion residue obtained from Cu/BaO_2_ (Figure 3) contain signals attributed to copper(II) oxide (signals at 602, 332 and 290 cm^−1^), as well as to copper(I) oxide (signals at 525, 496, 412 and 296 cm^−1^). The broad peak at 514 cm^−1^ corresponds to the bending vibration of hydroxyl in copper hydroxide [23]. The broad peaks are usually considered to arise from defects, poor crystallinity and size effect.

**Figure 4:**
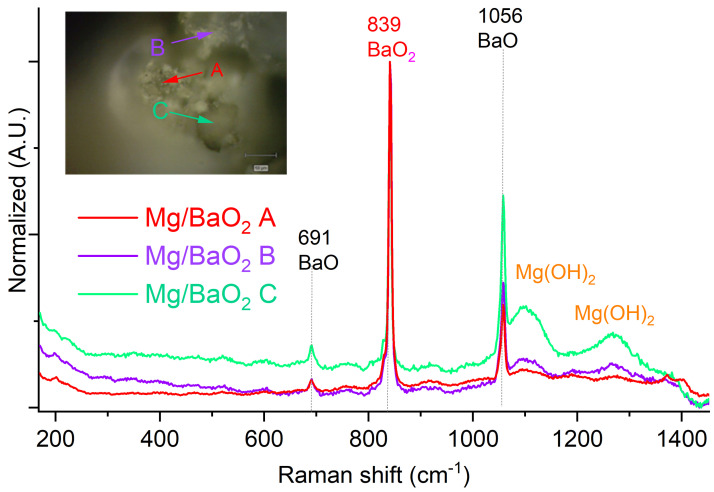
Raman spectra recorded for the combustion products of Mg/BaO_2_ PDC. Inset: Optical magnification of the sample with indicated points, for which Raman spectra were recorded.

In the case of Mg/BaO_2_, the Raman spectrum (Figure 4) additionally contains broad signals at 100 cm^−1^, 129 cm^−1^, 1095 cm^−1^, and 1290 cm^−1^, which are characteristics of a mixture of magnesium oxide and magnesium hydroxide. These signals matched those reported in the literature to a high extent [24].

**Figure 5:**
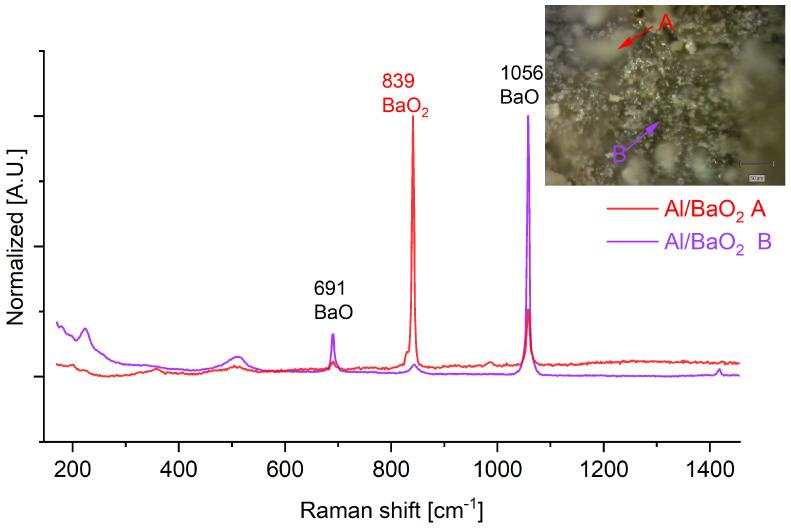
Raman spectra recorded for the combustion products of Al/BaO_2_ PDC. Insets: Optical magnification of the samples with indicated points, for which Raman spectra were recorded.

The Raman spectra (Figure 5 and Figure A7) of the post-combustion residues of the Al/BaO_2_ at an OB of +5% and 0% indicates the presence of only barium peroxide and barium oxide. In the case of combustion products of systems with negative OB values, are signals originating from the presence of alumina (signals at ~330, 505, 645 and 987 cm^−1^) observed.

**Figure 6:**
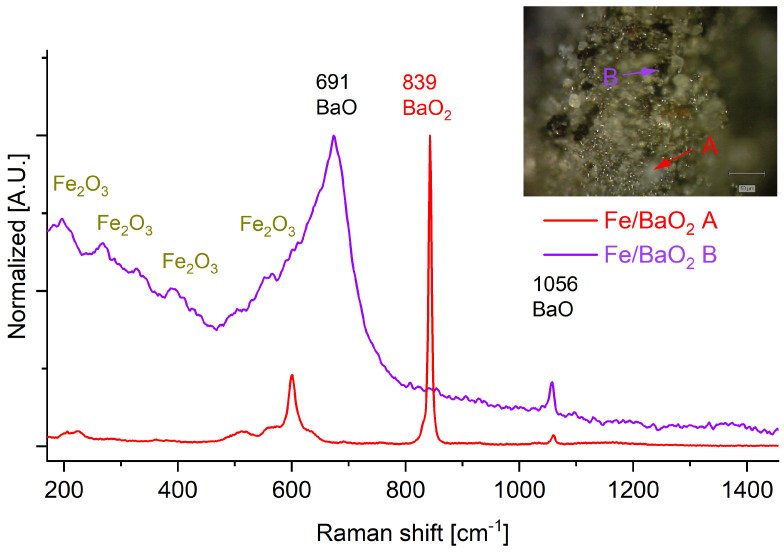
Raman spectra recorded for the combustion products of Fe/BaO_2_ PDC. Insets: Optical magnification of the samples with indicated points, for which Raman spectra were recorded.

The post-combustion residue of the Fe/BaO_2_ PDCs (Figure 6 contains Fe_2_O_3_ (strong resonant peaks at about 221, 287, 401, 493, and 641 cm^−1^ in the range of 200–800 cm^−1^ all correspond to the peaks for α-Fe_2_O_3_). In the spectrum of the combustion products of the Fe/BaO_2_-20 sample, signals at 634 cm^−1^ and at 1384 cm^−1^ indicate the presence of iron hydroxide oxide [25]. No appreciable presence of other iron oxides (e.g., FeO) is observed. It should be noted that many points in the investigated samples are seen in the optical magnifications as white particles. In the case of this particular PDC, these white particles are almost solely composed of unreacted barium peroxide.

In the case of the other PDC post-combustion residues, the white particles are also present. These particles, seen in the optical magnifications for, but are typically composed of a mixture of BaO (signals at 1026 and 691 cm^−1^) and unreacted BaO_2_ (signal at 839 cm^−1^, attributed to stretching oscillations of the peroxide bridge). The extensive presence of presence of unreacted barium peroxide, even in the case of products of combustion of PDCs exhibiting strongly negative oxygen balance values (OB of −20%) indicates that the combustion of BaO_2_-based PDCs follows a non-standard mechanism and that combustion is likely limited by the rate of decomposition of BaO_2_.

### 2.4. Combustion Mechanism

The first aspect relevant to the combustion of the investigated PDC formulations is that barium peroxide undergoes thermal decomposition at above 500 °C [26]. It is worth noting that the presence of impurities does not appear to significantly change the decomposition features of BaO_2_, as evidenced by DTA analyses reported in the literature [27].

The combustion of Cu/BaO_2_ takes place with the formation of both CuO and Cu_2_O (Figure 3), indicating that BaO_2_ is not a sufficiently active oxidising agent to readily oxidise copper. This is consistent with the observed residual BaO_2_ in the PDC post-combustion sample.The presence of Cu_2_O could be also related with CuO decomposition, and the mentioned compound is a intermediate product [28]. The presence of copper hydroxide may stem from the reactions of either of the two copper oxides with atmospheric moisture at high temperatures. Due to the low LCV of this PDC, these temperatures were maintained in the sampels for a prolonged time.

In the case of Mg/BaO_2_, reported DTA studies have found that the composition ignites at 540 °C, but shows a pre-ignition exothermic peak at 465 °C [29], an occurrence not found in that work for either of the PDC components. It should be noted that the melting point of Mg is only 650 °C [9], due to which it is expected that most of the combustion reaction takes place when magnesium is in the liquid state, potentially transitioning to the gas phase due to the low boiling point of Mg (1093 °C) [10]. This is in line with the observed fact that only upon compaction, the LCV can be measured using our device, as compaction would significantly decrease the mobility of the fluid fuel phase. The transition of Mg to the liquid or gas phases also explains the observed presence of Mg(OH)_2_ (Figure 4) in the post-combustion residues, as it would allow direct contact of Mg with atmospheric moisture, without the interference of the MgO layer typically forming on the surface of Mg grains in contact with oxygen.

The limited reactivity of BaO_2_ is again evidenced in the case of the Al/BaO_2_ (Figure 5 and Figure A7), as in the case of PDC samples with oxygen balance of +5% and 0%, where the amount of residual barium peroxide and of BaO is such that there are no Raman signals of aluminium oxides. Only in the case of PDC samples with negative oxygen balances, signals originating from alumina are observed, even though the signals of barium compounds still dominate the spectra.

**Figure A6:**
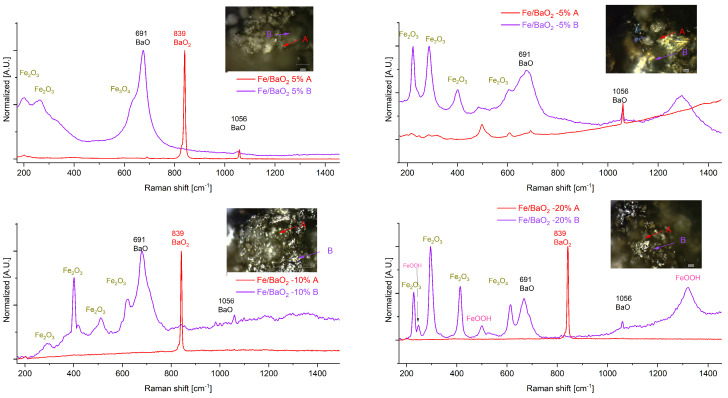
Raman spectra recorded for the combustion products of Fe/BaO_2_ PDC. Insets: Optical magnification of the samples with indicated points, for which Raman spectra were recorded.

**Figure A7:**
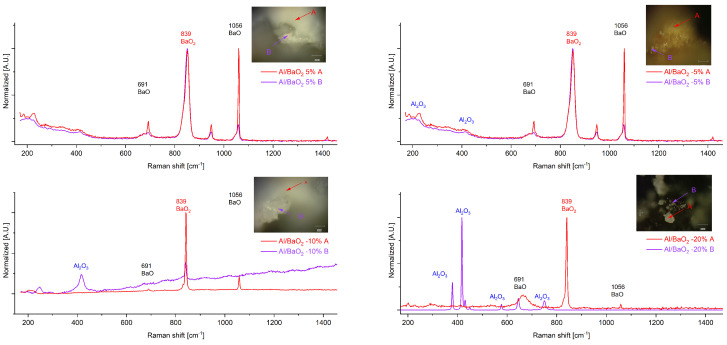
Raman spectra recorded for the combustion products of Al/BaO_2_ PDC. Insets: Optical magnification of the samples with indicated points, for which Raman spectra were recorded.

For Fe/BaO_2_, the main products of combustion are Fe_2_O_3_ and BaO (Figure 6 and Figure A6). Interestingly, in the case of the PDC with an OB of −5%, no barium peroxide signals are observed, contrary to all other samples of this PDC. The fact that compaction resulted in an increase in the LCV indicates, in conjunction with the high melting point of iron that most of the combustion reaction will either take place between two solid phases (Fe and BaO_2_) or between iron and the oxygen release due to the thermal decomposition of BaO_2_. In either case, the contact between the reagents is less intimate than if the fuel were molten or vaporised (as in the case of Mg or Al). This is the case even though the utilised iron was composed of the smallest grains of all the utilised fuels, which should have significantly promoted contact between the reagents. Due to this and the low net heat of combustion (Table 1) of this PDC, the removal of any microscopic air pockets due to combustion will help limit heat losses from the composition to the environment.

In no case have we observed the presence of the mixed oxides postulated in the literature (e.g., [18]). In contrast, in all cases, we have found evidence for the existence of residual or formation of new soluble barium compounds (BaO_2_ and BaO). This indicates that although BaO_2_ appears as a potentially useful oxidising agent, it cannot be considered “green”, as the post-combsution residues will eventually contaminate the environment, e.g., due to leaching.

## 3. Materials and Methods

### 3.1. Preparation of PDCs

The components (Table 5) were weighed so as to obtain 60 g of each mixture (Table 6). Thereafter, the compositions were mixed by brushing the composition through a 160 μm sieve five times and mixed with the cone method between brushing operations. The sieve-brush-mixing operation was performed to break up of particle agglomerates and to facilitate good mixing. After mixing, the compositions were stored in a dryer in 60 °C, in order to avoid absorbing moisture.

### 3.2. Material Characterisation

Friction and impact sensitivity were performed according to relevant international standards, using the Peters’ friction apparatus [21] and BAM Fallhammer apparatus [22], respectively.

Raman spectroscopy was performed using a Raman microscope (inVia Renishaw, Wotton-under-Edge, UK), which was equipped with a CCD detector and using red (633 nm) laser excitation. Spectra were recorded in a fixed range of 190–1450 cm^−1^. All measurements were made in a backscattering geometry using a 20× microscope objective with a numerical aperture value of 0.75, providing scattering areas of 1 μm^2^. Single-point spectra were recorded with 4 cm^−1^ resolution and 10 s accumulation times, with 10 spectra being acquired and averaged for each point.

The morphology and chemical composition of the post-combustion residues were investigated using a FEI Inspect S50 (FEI, Hillsboro, OR, USA) scanning electron microscope (SEM) equipped with an X-ray energy dispersive spectrometer (EDS) Octane Elect Plus (EDAX Inc., Mahwah, NJ, USA) with Apex Advanced software (Version 3.8.6.3964), which was used for the surface analysis, and an SEM Phenom ProX (Waltham, MA, USA). The basic SEM operation parameters were that the working distance was 10–11 mm, the acceleration voltages of the incident electron were 5 kV and 20 kV, the electronic beam spot size was 5 and the current intensity of the incident electronic beam was about 100 μA. The SEM-EDS images were recorded at 1000× magnification.

### 3.3. Determination of the Linear Combustion Velocity

**Figure 7:**
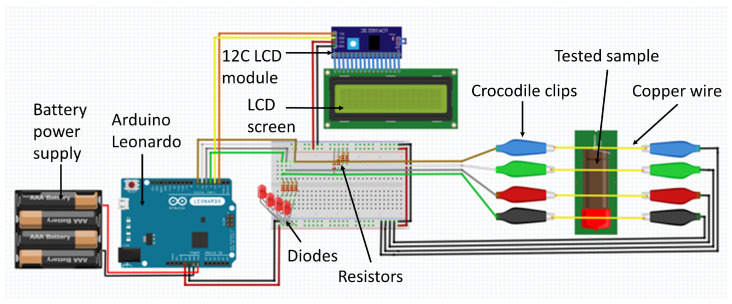
Schematic diagram of the linear combustion velocity measuring device.

Linear combustion velocity was determined using an electrical method. A diagram of the device is shown in Figure 7. The most important component of this device is the Arduino Leonardo board, which, based on the signals flowing through the crocodile clips and then the copper wires, calculates the combustion time of the samples and displays the results on the LCD screen. To make it possible to display the results on the LCD screen, an I2C LCD module was used, which allows direct data transfer from the Arduino board to the LCD display, using the I2C interface. The use of this component significantly simplified the operation of the LCD screen. The entire device has been enclosed in a compact, sealed enclosure to protect the individual components of the device from the negative effects of combustion the tested materials. Furthermore, for this purpose, we used a battery, so that the device in its entirety could be enclosed under the fume hood where the combustion of the material was carried out. The correct connection of four copper probes to the measuring device is indicated by the illumination of four LEDs, in which each corresponds to one measuring point. The end of the measurement is signaled by the last diode shutting down, due to the last circuit formed by the copper wire probes being broken.

The delay elements were produced using 15 cm cellulose tubes. The tubes had an outer diameter of 8 mm and an inner diameter of 7 mm. The testing tubes were prepared in such a way that four pieces of copper wire were drawn through the plastic tube, approximately 2 cm apart. The initial distance to stabilise the combustion velocity between the ignition point and the first probe was equal to approximately 3 cm. The tubes were then loosely backfilled with the tested composition. The initiating source was a C56 chlorate fuse igniter, for each of the measurements.

To briefly describe the effect of compaction on combustion process, the linear combustion velocity test was repeated for the four PDC samples, whose composition corresponded to an oxygen balance values of O%. The test again used plastic tubes with an inner diameter of 7 mm and a length of 15 cm, through which four copper wires were passed, 3 cm apart, connected to the described device. The initial distance to stabilise the combustion velocity between the ignition point and the first probe was too equal to approximately 3 cm. The compositions were compacted by gently tamping and vibrating to achieve the desired density (approximately 30% TMD). The initiating source was again a C56 chlorate fuse igniter.

## 4. Conclusions

The presented results show that all four investigated compositions can be viable as PDCs, with the choice of metallic fuel and quantitative composition (expressed by the oxygen balance value) of the PDC having a significant effect on their combustion parameters. The observed deviations, in terms of both mechanical sensitivity and combustion velocity, from expected trends are indicative that the mechanism underlying the combustion of these systems is not straightforward.

The most likely hypothesis, particularly based on the analysis of SEM-EDS mappings and Raman spectra, is that combustion of these PDCs is limited by the rate of decomposition of BaO_2_. This is evidenced by the presence of unreacted barium peroxide in the post-combustion residues, even in the case of PDCs, whose contents correspond to strongly negative oxygen balance values (OB of −10 or −20%).

The increase in the observed combustion velocity upon the use of a more energetic fuel (i.e., replacement of iron for aluminium or magnesium) indicates that the issue of the limited rate of BaO_2_ decomposition may be overcome through increasing the combustion temperature of the PDCs. Consequently, BaO_2_ is not viable as a “green” oxidising agent, at least in the case of the investigated PDC formulations. Moreover, if it is found to be viable, it will likely be limited to compositions containing highly reactive and energetic fuels, such as Al or Mg.

## Figures and Tables

**Table 2:** Sensitivity parameters for the investigated PDCs.

	PDC	Fe/BaO_2_		Al/BaO_2_	
OB		FS	IS	FS	IS
+5%		24	>50	20	>50
0%		32	>50	12	>50
−5%		20	>50	10	>50
−10%		28	>50	10	>50
−20%		30	40	10	>50

Oxygen balance. The OB was calculated assuming complete oxidation of the fuels; sensitivity to friction, as per [21]; sensitivity to impact, as per [22].

**Table 3:** Linear combustion velocity (LCV) of the investigated uncompacted PDC samples.

	PDC	LCV [cm/s]	
OB		Fe/BaO_2_	Al/BaO_2_
+5%		0.117 ± 0.057 (20% TMD)	2.702 ± 0.315 (24% TMD)
0%		0.226 ± 0.069 (21% TMD)	2.220 ± 0.251 (25% TMD)
−5%		0.137 ± 0.042 (23% TMD)	2.364 ± 0.236 (21% TMD)
−10%		0.027 ± 0.008 (21% TMD)	2.344 ± 0.218 (23% TMD)

Oxygen balance. The OB was calculated assuming complete oxidation of the fuels; linear combustion velocity, presented as value ± standard deviation for n = 5 samples.

**Table 4:** Linear combustion velocity (LCV) of PDC samples compacted to approximately 30% TMD.

	Parameter	LCV [cm/s] ^a^	Density [% of TMD]
PDC			
**Fe/BaO_2_**		0.330 ± 0.059	34
**Al/BaO_2_**		1.258 ± 0.394	33
**Mg/BaO_2_**		5.533 ± 0.929	32
**Cu/BaO_2_**		0.227 ± 0.054	32

^a^—Linear combustion velocity, presented as value ± standard deviation for n = 5 samples.

**Table 5:** Materials used in this work.

Chemical	Purity Grade	Source	Grain Size
Barium peroxide	>95%	Thermo Fisher Scientific (Waltham, MA, USA)	<500 µm
Aluminium	>95%	POCH S.A (Gliwice, Poland)	105 µm
Iron	>95%	POCH S.A. (Gliwice, Poland)	12 µm
Magnesium	>95%	POCH S.A (Gliwice, Poland)	89 µm
Copper	>95%	Selkat	63 µm

Average particle size distribution of the raw materials was dconfirmed using Frith GmbH Analysette 22 laser particle analyser.

**Table 6:** Components of investigated PDCs.

	PDC	Fe/BaO_2_		Al/BaO_2_	
OB		BaO_2_ wt.%	Fe wt.%	BaO_2_ wt.%	Al wt.%
+5%		91.51	8.49	95.48	4.52
0%		81.97	18.03	90.39	9.61
−5%		72.34	27.66	85.32	14.68
−10%		62.89	37.11	80.23	19.77
−20%		43.82	56.18	70.07	29.93
	**PDC**	**Mg/BaO_2_**		**Cu/BaO_2_**	
**OB**		**BaO_2_ wt.%**	**Mg wt.%**	**BaO_2_ wt.%**	**Cu wt.%**
0%		87.45	12.55	72.71	27.29

The oxygen balance (OB) was calculated assuming complete oxidation of the fuels.

**Figure A2:**
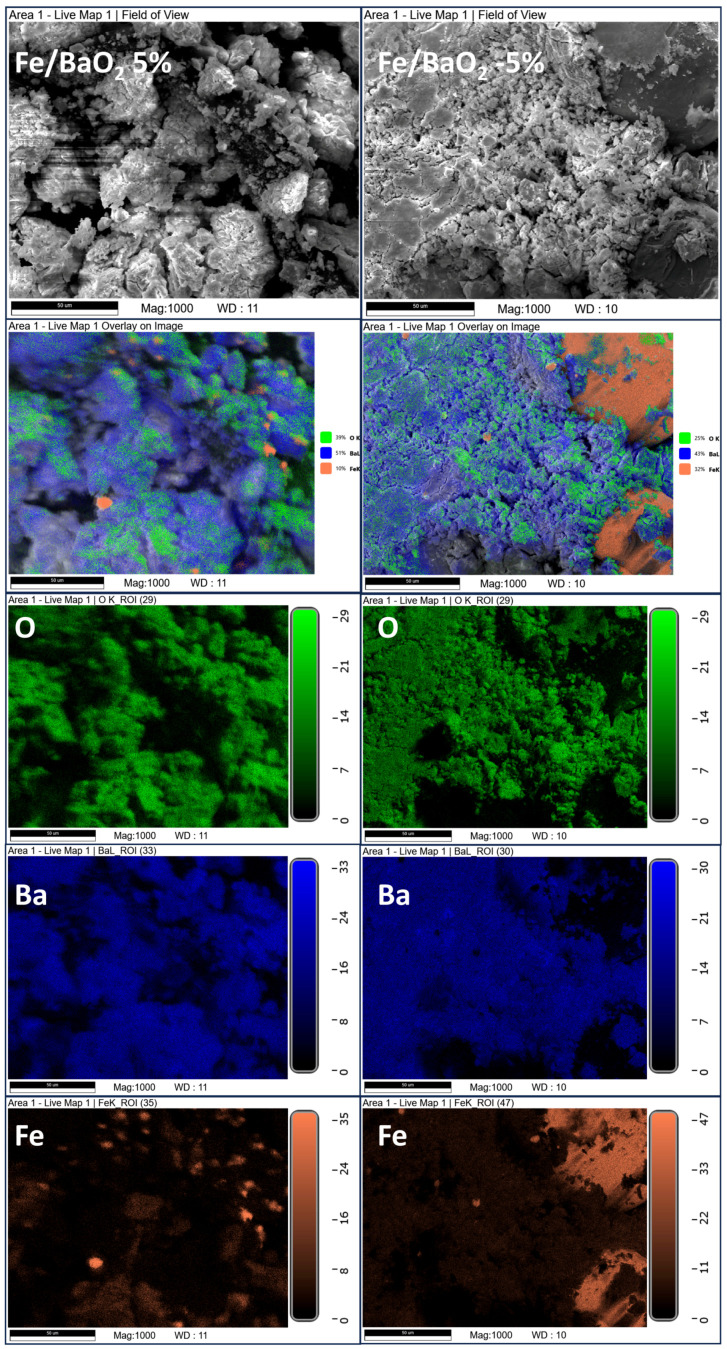
SEM images and EDS-based elemental mapping overlays for post-combustion residues of the Fe/BaO_2_ PDC produced to have an oxygen balance of +5% and −5%.

**Figure A3:**
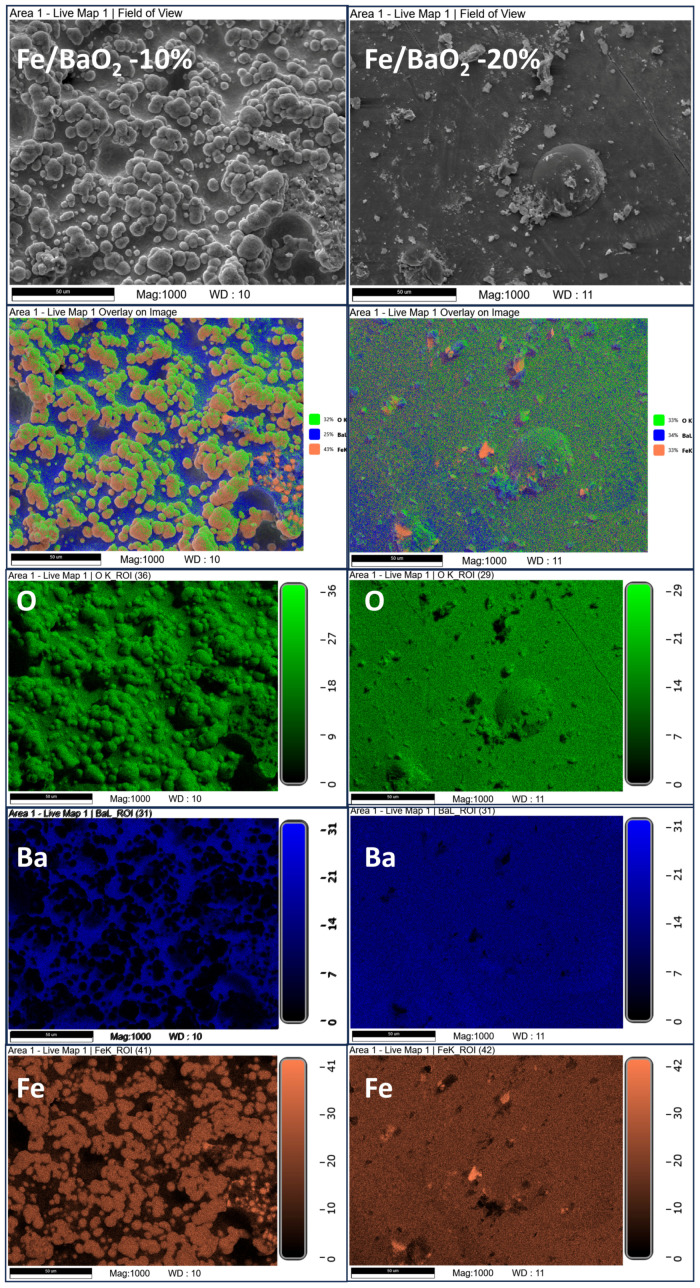
SEM images and EDS-based elemental mapping overlays for post-combustion residues of the Fe/BaO_2_ PDC produced to have an oxygen balance of −10% and −20%.

## Data Availability

Data is available from the authors on request.
